# The therapeutic effect and targets of cellulose polysaccharide on coronary heart disease (CHD) and the construction of a prognostic signature based on network pharmacology

**DOI:** 10.3389/fnut.2022.986639

**Published:** 2022-10-10

**Authors:** Lang Liu, Yundi Zhang, Yuxin Du, Haoyue Li, Mingzhao Wang, Jianfeng Lv

**Affiliations:** ^1^Department of Cardiology, General Hospital of Ningxia Medical University, Yinchuan, China; ^2^National Cancer Center/National Clinical Research Center for Cancer/Cancer Hospital, Chinese Academy of Medical Sciences and Peking Union Medical College, Beijing, China; ^3^Department of Cardiology, Shanghai Institute of Cardiovascular Diseases, Zhongshan Hospital Fudan University, Shanghai, China; ^4^Department of Cardiology, Affiliated RenHe Hospital of China, Second Clinical Medical College, Three Gorges University, Yichang, China

**Keywords:** polysaccharide, cellulose, CHD, network pharmacology, molecular docking

## Abstract

Cellulose is the first rich biological polysaccharide in nature and has many excellent properties, so it is being developed as a variety of drug carriers. Moreover, applications in drug delivery, biosensors/bioanalysis, immobilization of enzymes and cells, stem cell therapy, and skin tissue repair are also highlighted by many studies. Coronary heart disease, as one of the diseases with the highest incidence, is urgent to enhance the survival outcome and life quality of patients with coronary heart disease, whereas the mechanism of cellulose's interaction with the human body remains unclear. However, the mechanism of cellulose's interaction with the human body remains unclear. We obtained 92 genes associated with cellulose and coronary heart disease through the intersection of different databases. Ten key genes were identified: HRAS, STAT3, HSP90AA1, FGF2, VEGFA, CXCR4, TERT, IL2, BCL2L1, and CDK1. Molecular docking of the 10 genes revealed their association with their respective receptors. Analysis by KEGG and GO has discovered that these related targets were more enriched in metabolic- and activation-related functions, which further confirmed that cellulose polysaccharides can also interact with cardiovascular diseases as molecules. In the end, we screened out six key genes that were more associated with the prognosis (CDK1, HSP90AA1, CXCR4, IL2, VEGFA, and TERT) and constructed a signature, which has a good predictive effect and has significant statistical significance. Our study is the first study to explore the interaction targets of cellulose and CHD and to construct a prognostic model. Our findings provide insights for future molecular design, drug development, and clinical trials.

## Introduction

Coronary heart disease refers to the accumulation of some lipid substances similar to atherosclerosis in the intima of the artery and becomes white plaques, which gradually increase to cause arterial stenosis, blocked blood flow, resulting in heart ischemia, and angina pectoris (1, 2). With the improvement of people's living standard, coronary heart disease has growingly become a global health problem and the main cause of global morbidity. Many risk factors contribute to coronary heart disease, including hypertension, hyperlipidemia, aging, and diabetes (3, 4). Coronary heart disease can be treated through drug treatment, surgical treatment, PCI treatment, and so on Stewart et al. (5). The prognosis varies with the site and severity of the lesion, the rate of progression of vascular stenosis, the damage of the affected organs, and the presence or absence of complications.

Polysaccharides, being formed by glycosidic bonds of long carbohydrate molecules with monosaccharide units, (6, 7) exist widely in animals, plants, and microorganisms and are easy to obtain and intently relative to tremendous kinds of physiological functions. It is one of the four fundamental substances that constitute life (8, 9). Studies have shown that polysaccharides have anti-virus, anti-tumor, anti-oxidation, immune regulation, and other biological activities. (10–12). Previous researches have demonstrated that polysaccharides have a significant efficacy on the treatment of coronary heart disease (6). Especially in Chinese traditional medicine, cellulose polysaccharides are very rich, and many of them are used in the treatment of coronary heart disease. Bacterial cellulose (BC), one of the polysaccharides, has attracted great interest in medicine, pharmacy, and other related fields due to its inherent physical, mechanical, and biological properties (13). Its structural characteristics provide an ideal environment for the development of composite materials (14, 15). Compared with plant cellulose, bacterial cellulose (BC) has the characteristics of high purity, high water retention, and biocompatibility (16, 17). Cellulose nanocrystals, a nanostructure of cellulose material as a new research direction of biomedical resources, with excellent performance results, such as sustainability, biodegradability, and biocompatibility, have become a new field of polysaccharide treatment in some diseases (18–20). At present, studies have shown that nanocellulose can be used as a tumor drug carrier and can play a role in the treatment of tumors. However, its therapeutic effect on coronary heart disease is still unclear.

At the macrolevel, the intake of cellulose can regulate the intestinal microbiota of the human body (21). The intestinal flora, through its metabolites, participates in mediating the metabolism of blood cholesterol or uric acid, even taking part in oxidative stress, inflammatory response, and other basic metabolic processes, which may possibly lead to the progress of atherosclerosis and coronary heart disease. Moreover, the intake of cellulose helps to regulate the level of blood lipid and thus may play a role in the treatment of coronary heart disease. Cellulose is the richest polysaccharides on earth and has many excellent characteristics, including low expense, brilliant biodegradability, and good biocompatibility (22). This makes cellulose a brilliant material that can be potentially used for creating nano-drug delivery systems (nano-DDs). A study published by Lin, Dai et al. introduces and discusses some significant advances in the formulation of cellulose-based prodrugs and nanoparticles (23–26). Microscopically, nanocellulose has high medical value and can be used as the management direction of coronary heart disease in future (27). Nanocellulose is an exogenous substance in the human body, and its exact toxicity and how it interacts with human tissues are not clear yet. Therefore, it should be studied and examined in detail before any biomedical application that requires direct contact with human cells. Nowadays, nanomaterials have gradually become an important part of new drugs and medical materials, and nanocellulose has the most promising application prospects in biomedical fields such as regenerative medicine, tissue engineering, and controlled drug delivery. Nanocellulose is considered to be a biocompatible nanomaterial, which is relatively safe for biomedical applications.

Therefore, it is very important to find relevant targets and molecular pathways as the basis for research. We hypothesized that pathways related to cell membrane signaling, cell stability, and drug response were most affected. Studies have reported that immune responses are closely related (28). However, more research is needed to prove this hypothesis. In this study, a series of analyses were conducted on the data from public databases to find the most important targets and carry out enrichment analysis of relevant physiological processes, so as to explore the influence of cellulose on coronary heart disease at the level of certain molecular targets.

## Materials and methods

### Data collection

In the cause of finding the targets of cellulose, two online databases for targets of medications' searching and screening were applied: SwissTargetPrediction (http://www.swisstargetprediction.ch/) and Similarity ensemble approach (SEA) search server (https://sea.bkslab.org/). For selecting the query molecule in the online database named SwissTargetPrediction, the standardized screening threshold of taking the protein as target was set to be >0. In the SEA database, we screened targets for the cellulose and recorded those with E-values of <10–5 for cellulose-target analysis. The targets for coronary heart disease (CHD) were obtained from the following five databases, including DisGeNET database (https://www.disgenet.org/), the DrugBank database (https://www.drugbank.ca/), GeneCards database (https://www.genecards.org/), MalaCards database (https://www.malacards.org/), and Online Mendelian Inheritance in Man database (OMIM, https://www.omim.org/). Afterward, a CHD-related gene set was generated by uniting the results from these databases.

The previously published datasets downloaded from NCBI Gene Expression Omnibus (NCBI-GEO, https://www.ncbi.nlm.nih.gov/geo/) database (i.e., GSE35182, GSE62867, GSE169256, GSE186019, GSE190475, GSE194154, GSE194155, GSE194156, and GSE198885) were screened out to onwards acquire data of gene expression. Supported by the GEO2R (http://www.ncbi.nlm.nih.gov/geo/geo2r/) analysis, genes that are diversely expressed with the cutoff criteria of |Log2FC| more than one and adjusted *P*-value <0.05 were selected in comparison with normal heart tissues.

### Venn diagram construction

Venn diagram was established by utilizing VENNY 2.1 (https://bioinfogp.cnb.csic.es/tools/venny/) according to the intersection of the target genes of CHD and cellulose. Ninety-two intersectant targets were obtained following inputting 8,051 differentially expressed genes in CHD and 146 target genes of cellulose. The coincident target genes between both cellulose and CHD were displayed in the overlapping domain after amalgamation and striking out the duplicates.

### Establishment of protein–protein interaction (PPI) network

The network of PPI cellulose and CHD reclosing targets in Venn diagram was constructed by utilizing the database STRING (https://string-db.org/). The genes that met the interaction grade exceeding the threshold (≥0.4) were sorted for the generation of PPI network. The PPI network was depicted by Cytoscape 3.8.2 software (https://cytoscape.org/), and a highly connected sub-networks were screened out by the Molecular Complex Detection (MCODE) plugin (version 1.5.1) of Cytoscape with a threshold cutoff being equaled to two, cutoff of node score being equaled to 0.2, K-core being equaled to two, and max depth being equaled to 100.

### Analysis of gene ontology (GO) and kyoto encyclopedia of genes and genomes (KEGG)

Enrichment analyses by GO and KEGG were performed to pursue the biological capabilities of the overlapping target genes by utilizing the online Metascape (http://metascape.org/gp/index.html#/main/step1). Bioinformatics (http://www.bioinformatics.com.cn) was used to visualize the representative enriched terms. The results include cellular component (CC), biological process (BP), and molecular function (MF) discovered by GO analysis and key signaling pathways generated by KEGG analysis that were finally presented in bar and bubble graph.

### Molecular docking analysis

Molecular docking analyses of cellulose and the major relevant targets were performed to validate the reliability of the prediction results. The two-dimensional (2D) structure of cellulose was downloaded and obtained from the database named PubChem (https://pubchem.ncbi.nlm.nih.gov/). With the software OpenBabel 2.4.1, those data were converted to the format of mol2. The proteins of relevant receptors encrypted by the opted target genes were searched in the UniProt database (http://www.uniprot.org/). In addition, 3D compositions of the protein receptors were acquired from the Protein Data Bank (PDB) database (https://www.rcsb.org/). PyMol software and AutoDockTools 1.5.6 software were used to perform the dehydration and hydrogenation process for the protein molecules, respectively. Finally, cellulose and protein receptors were docked by utilizing AutoDock Vina software.

### Prognosis analysis

Six genes were normalized by using Log2 transformation. We conducted minimum absolute shrinkage, LASSO regression, and multivariate Cox regression analysis with R package “glmnet” in sequence. A signature based on these six genes was conducted. With the usage of “survival” R package, Kaplan–Meier survival analysis evaluating the survival differences between the high- and low-risk groups was finished. Time-dependent receiver operating characteristic (ROC) curves were used to evaluate the performance of genetic risk models. Moreover, “survivalROC” R packages were used to examine the prognosis prediction efficiency and other clinical characteristics. An analysis named Cox regression was applied to assess the independent prognostic value of clinical characteristics. With the aim of estimating the likelihood of survival outcomes, a survival map by nomogram was constructed using R package named “rms” according to risk score and clinical features. It was also examined by analysis of multivariate Cox regression. The relative function of nomogram was appraised by C index, ROC, and calibration chart.

## Results

### Target genes of cellulose and CHD

**Figure 1:**
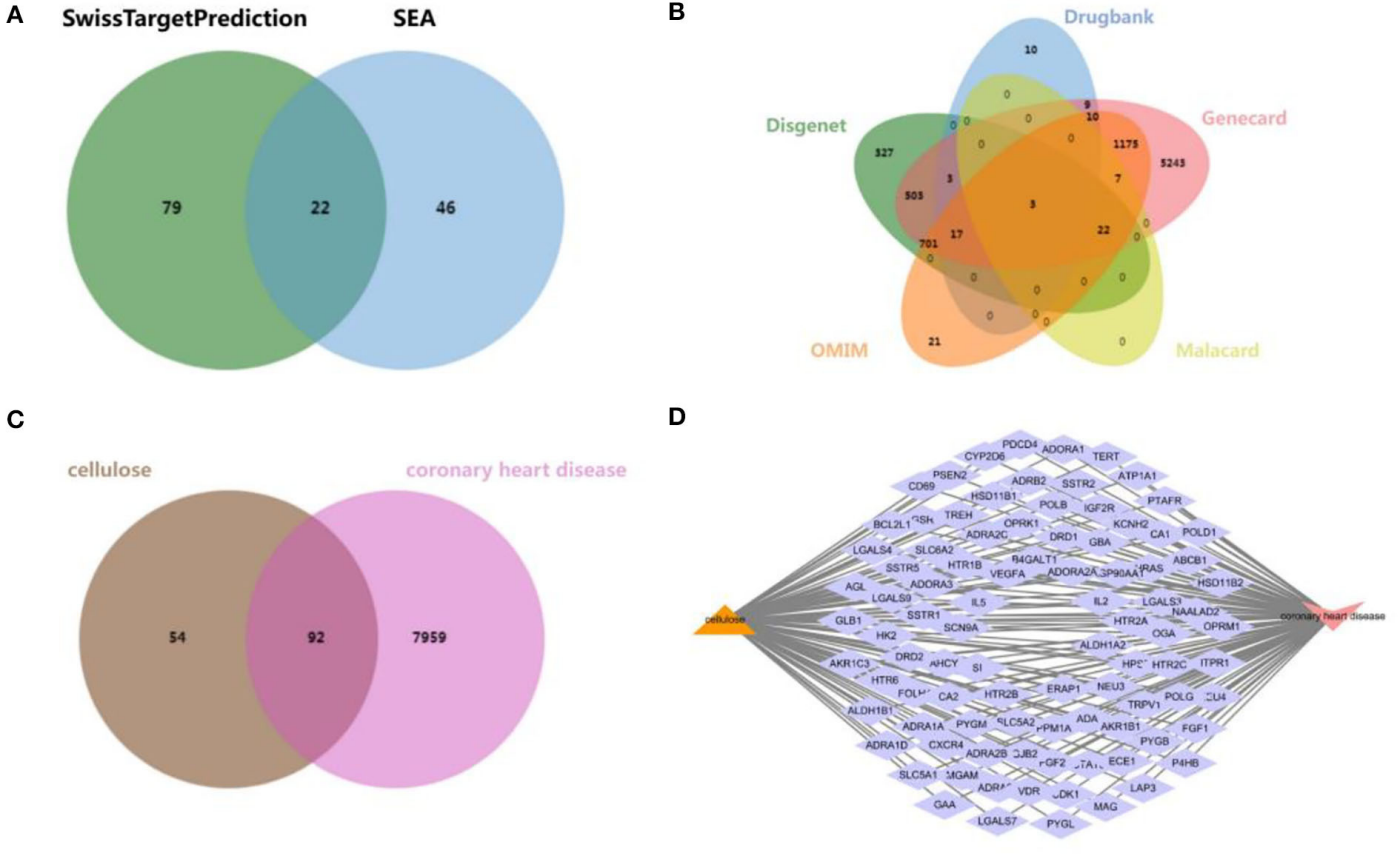
Exploration of CHD and cellulosic polysaccharide-related targets. **(A)** Venn diagram from SwissTargetPrediction and SEA database. **(B)** Venn diagram from DisGeNET database, DrugBank database, GeneCards database, MalaCards database, and OMIM database. **(C)** Venn diagram of the interactive targets of cellulose and CHD. **(D)** Network diagram of the intersectant targets.

An overall 147 cellulose-related target genes were obtained from SwissTargetPrediction and SEA search server after removing duplication and combining the results (Figure 1A). A total of 1,576, 52, 7,693, 32, and 1,956 targets of CHD were collected from DisGeNET database, DrugBank database, GeneCards database, MalaCards database, and OMIM database, respectively, and a sum of 8,051 CHD-affiliated genes were acquired after duplicate target elimination and the result combination (Figure 1B). Furthermore, to reveal the interactive targets of cellulose and CHD, a Venn diagram was established to show the overlapped part of cellulose targets and CHD-affiliated genes (Figure 1C) and 92 intersectant genes were finally obtained (Figure 1D).

### PPI network and highly related subnetwork

**Figure 2:**
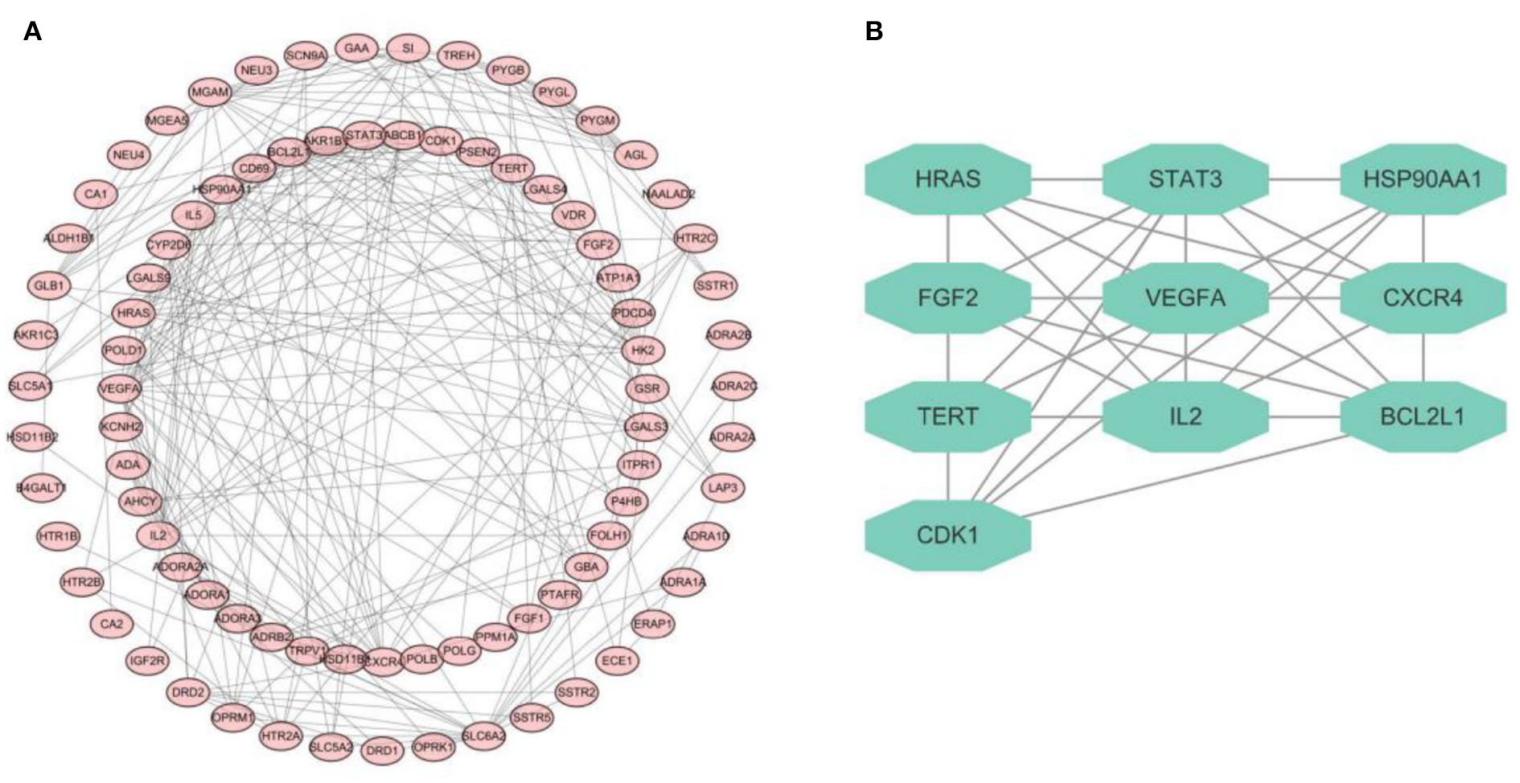
PPI network diagram. **(A)** PPI network of 92 targets derived from STRING database. **(B)** Network diagram of 10 key targets.

The network of PPI derived from database named STRING was constructed to explore the complex interactions among these 92 intersectant targets that were considered to be the interactive targets of cellulose and CHD. The target proteins and the interactions of these proteins are denoted by the nodes and edges, respectively, and the number of stria in the network of PPI indicated the level of correlations and target ranks (Figure 2A). Moreover, a key subnetwork composed of 10 target genes (i.e., HRAS, STAT3, HSP90AA1, FGF2, VEGFA, CXCR4, TERT, IL2, BCL2L1, and CDK1) was established as well by utilizing MCODE plugin of Cytoscape (Figure 2B). We found that these targets have a strong interaction with each other and speculated that they may collectively play some role.

### GO and KEGG enrichment analysis and molecular docking

**Figure 3:**
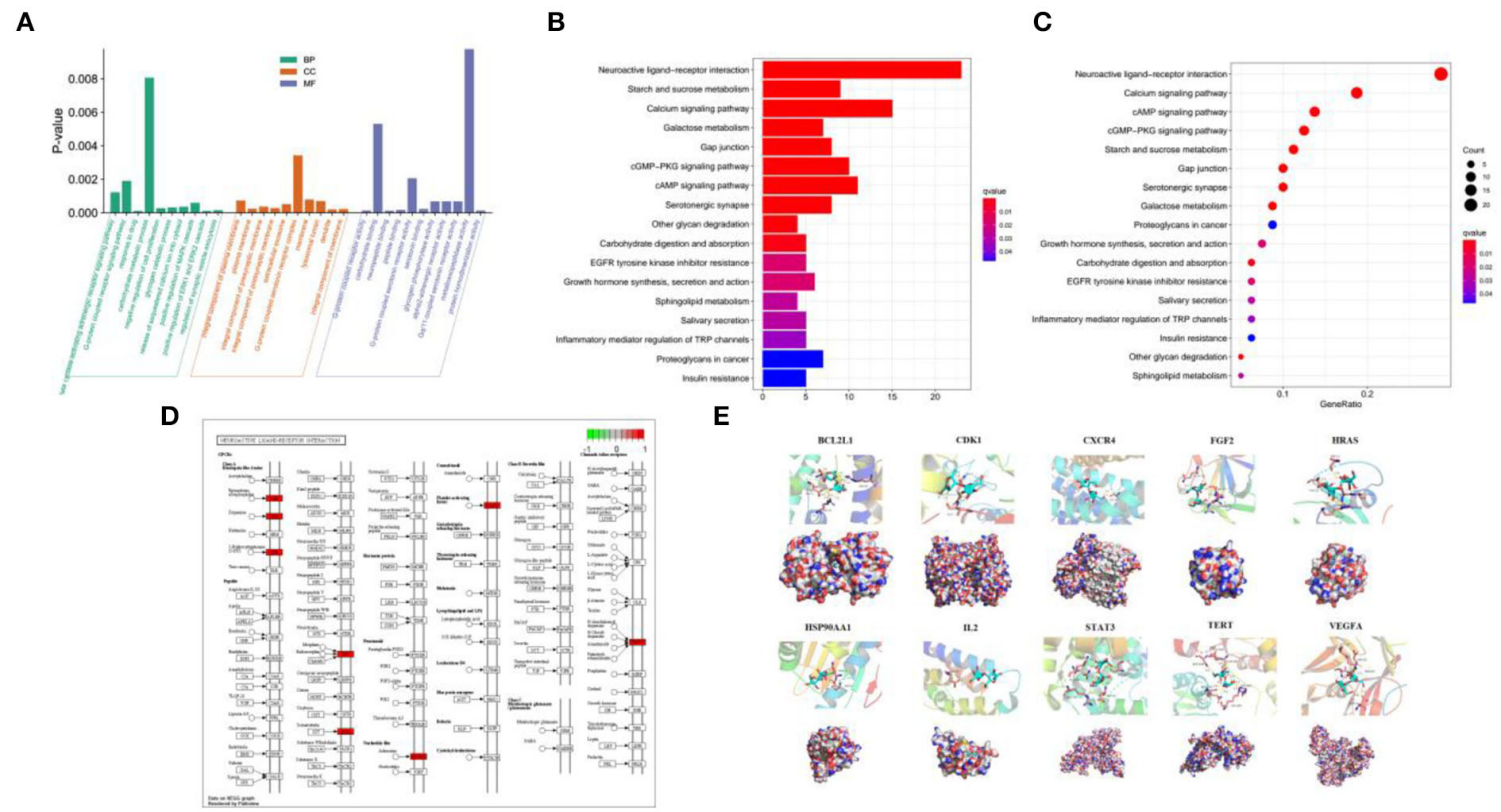
Analysis of GO and KEGG enrichment and molecular docking. **(A)** GO analysis of the 92 overlapping genes. **(B,C)** Analysis of KEGG among the 92 overlapping genes. **(D)** Signaling mapping of the overlapping genes. **(E)** Molecular docking of the 10 key targets.

The aforementioned 92 overlapping genes undergone the GO enrichment analysis, and the most significant 10 catalogs of biological process (BP), cellular component (CC), and molecular function (MF) were clarified, respectively (Figure 3A). The results of GO analysis suggested that cellulose affects CHD through various aspects, especially response to drug (BP), positive regulation of ERK1 and ERK2 cascade (BP), dendrite (CC), integral component of membrane (CC), neuropeptide binding (MF), and G-protein coupled receptor activity (MF).

KEGG enrichment analysis was used to analyze the 92 intersectant genes as well for a further investigation of the underlying pathways of cellulose influencing CHD. The bar and bubble chart illustrated the top 17 significant results (Figures 3B,C), and the map showed the interaction of neuroactive ligand–receptor (Figure 3D). KEGG analysis indicated that the interaction among neuroactive ligand–receptors, the pathways of calcium signaling, pathways of cAMP signaling, pathways of cGMP-PKG signaling, and metabolism of starch and sucrose where the 92 targets were enriched could be the potential mechanisms of cellulose affecting CHD. Besides, gap junction, serotonergic synapse, and galactose metabolism may also participate in the regulation of CHD by cellulose.

The ten target gene-encoded proteins in PPI subnetwork, including HRAS, STAT3, HSP90AA1, FGF2, VEGFA, CXCR4, TERT, IL2, BCL2L1, and CDK1, were selected to carry out molecular docking analyses with cellulose, respectively. The outcomes from molecular docking demonstrated that the cellulose has the capabilities to enter and connect the active pocket of all selected receptors (Figure 3E). The docking score of the 10 receptors with cellulose is shown in Table 1.

**Table 1:** Results of molecular docking.

**Receptor name**	**Ligand name**	**Scores**
BCL2L1	cellulose_config_out1.pdbqt	−7.3
CDK1	cellulose_config_out1.pdbqt	−7.4
CXCR4	cellulose_config_out1.pdbqt	−6.4
FGF2	cellulose_config_out1.pdbqt	−5.6
HRAS	cellulose_config_out1.pdbqt	−8.5
HSP90AA1	cellulose_config_out1.pdbqt	−7.2
IL2	cellulose_config_out1.pdbqt	−5.6
STAT3	cellulose_config_out1.pdbqt	−6.8
TERT	cellulose_config_out1.pdbqt	−7.0
VEGFA	cellulose_config_out1.pdbqt	−7.2

### Survival analysis and construction of prognostic model

**Figure 4:**
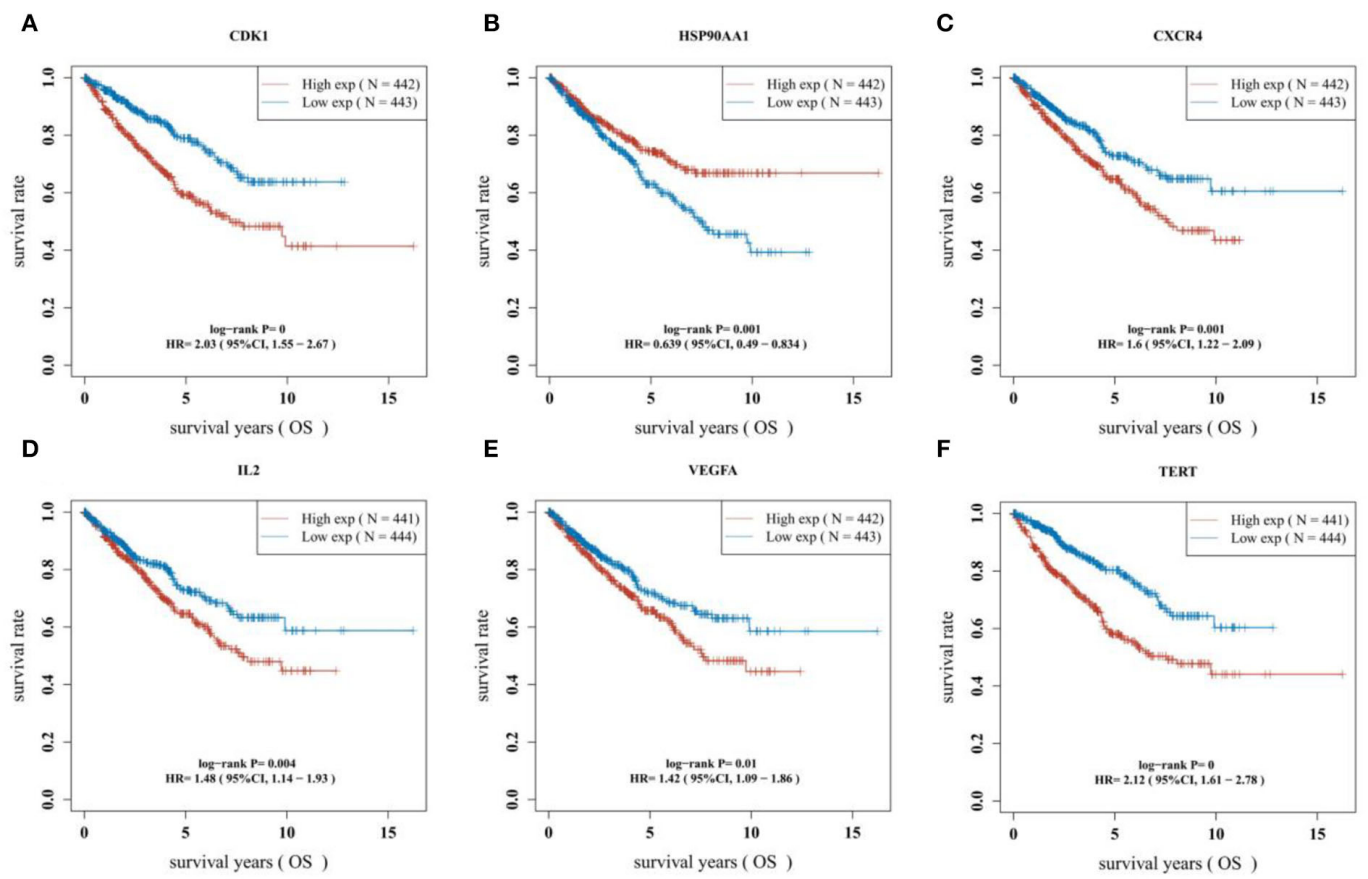
Difference of survival outcomes in CHD patients. **(A)** Patients with high expression of CDK1 have a lower survival rate (*P* < 0.0001, HR = 2.03, 95%CI: 1.55–2.67). **(B)** Patients with high expression of HSP90AAI have a better survival rate (*P* = 0.001, HR = 0.639, 95%CI:0.49–0.834). **(C)** Patients with high expression of CXCR4 have a lower survival rate (*P* = 0.001, HR=1.6, 95%CI:1.22–2.09). **(D)** Patients with high expression of IL2 have a lower survival rate (*P* = 0.004, HR = 1.48, 95%CI:1.14–1.93). **(E)** Patients with high expression of VEGFA have a lower survival rate (*P* = 0.01, HR = 1.42, 95%CI:1.09–1.86). **(F)** Patients with high expression of TERT have a lower survival rate (*P* < 0.0001, HR=2.12, 95%CI:1.61–2.78).

**Figure 5:**
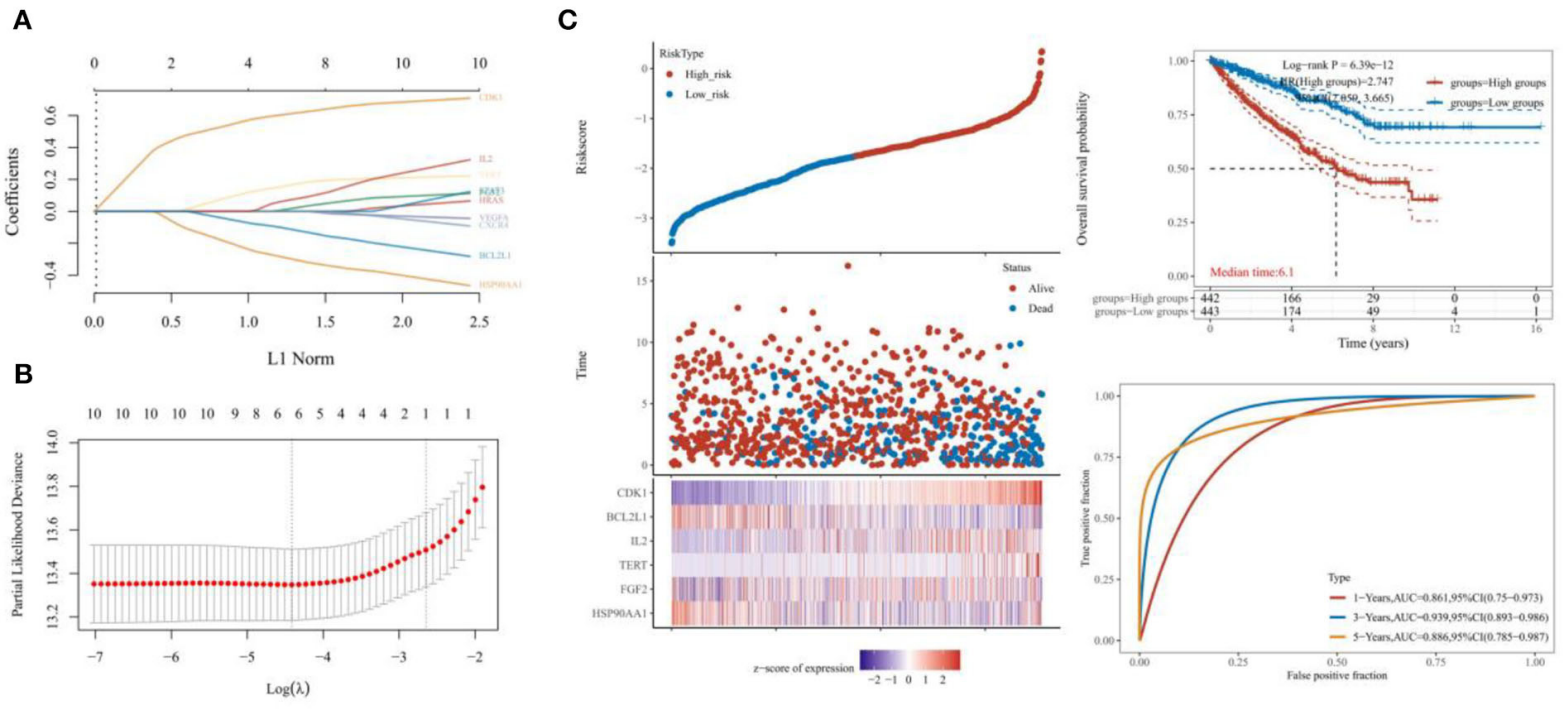
Efficacy test of six-gene prognostic signature. **(A,B)** Analysis by LASSO Cox regression and the partial likelihood deviance of the targets. **(C)** The Kaplan–Meier survival curve indicating the overall survival probability and the AUCs for 1, 3, and 5 years.

**Figure 6:**
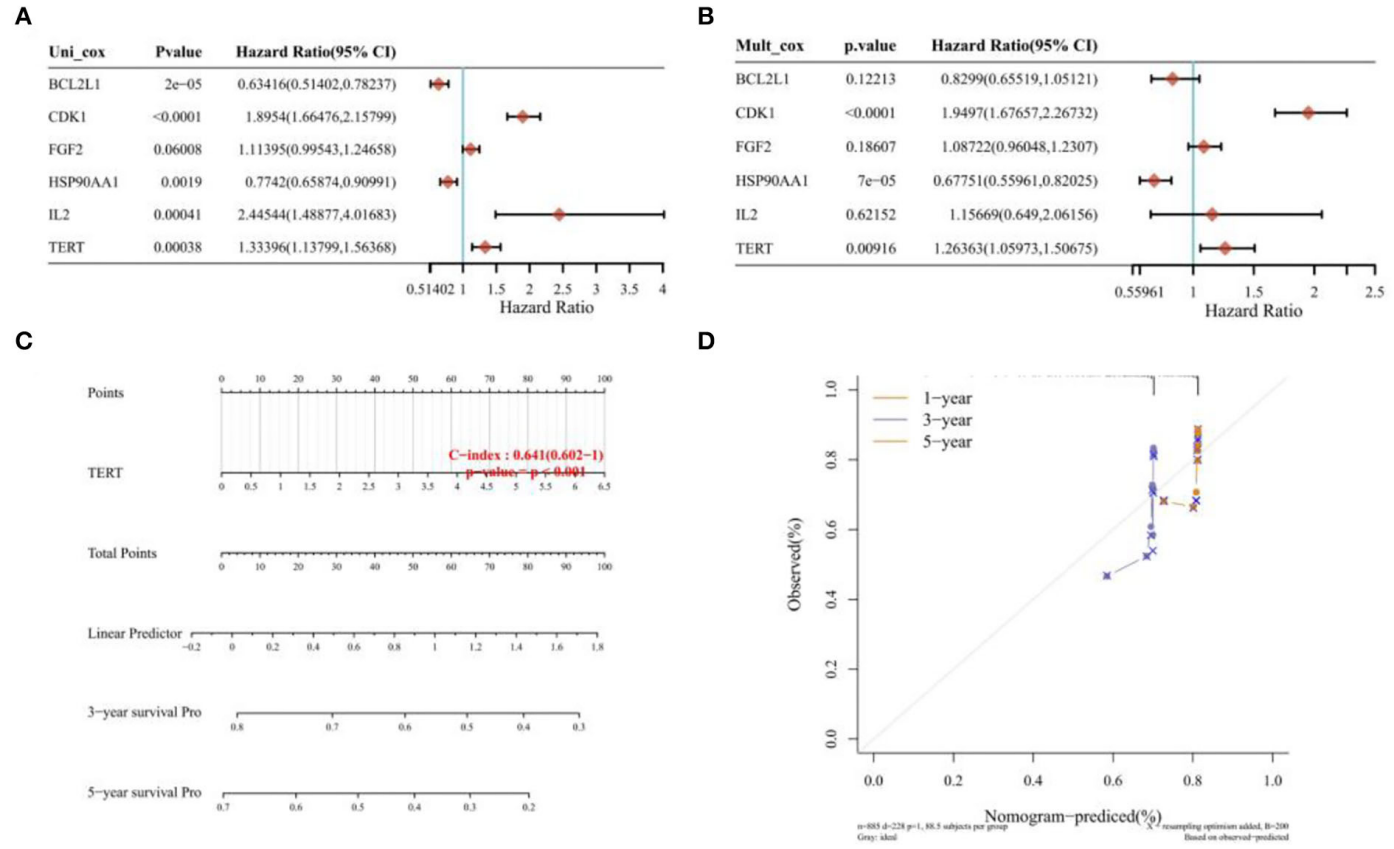
Univariate regression, multivariate regression, and nomograph. **(A)** Univariate Cox regression analyses of the expression of BCL2L1, CDK1, HSP90AA1, IL-2, and TERT of CHD patients. **(B)** Multivariate cox regression analyses of the expression of BCL2L1, CDK1, HSP90AA1, IL-2, and TERT of CHD patients. **(C)** Nomogram established by utilizing TERT. **(D)** Calibration plot of 1-year, 3-year, and 5-year survival.

The survival curves were established following analysis of the data from GEO database. A total of 442 heart tissues with high expression of six core genes and 443 with low expression were analyzed. The CHD patients with high expression of CDK1, CXCR4, IL2, VEGFA, and TERT showed a significant lower five- and ten-year survival rate, whereas the high expression of HSP90AA1 improved the survival rate of CHD patients (Figures 4A–F). These results indicated that CDK1, HSP90AA1, CXCR4, IL2, VEGFA, and TERT were potential targets for CHD intervention, and HSP90AA1 was a possible protective factor for CHD. Furthermore, analysis of LASSO Cox regression was performed to investigate the acquaintance among the ten core target genes from PPI subnetwork and the survival outcomes for CHD patients (Figures 5A,B), and six targets were sorted to establish the signature of the prognosis. The risk score was computed by the following formula: Risk score= (−0.3067) ^*^HSP90AA1 + (0.0239) ^*^FGF2 + (0.1721) ^*^TERT + (0.0799) ^*^IL2 + (−0.1204) ^*^BCL2L1 + (0.617) ^*^CDK1. According to the distribution of risk score, the patients with CHD were divided into high-risk groups and low-risk groups. The curve from analyzed Kaplan–Meier survival indicated that the overall survival probability of high-risk group was inferior to the low-risk group, and the AUCs for 1, 3, and 5 years were 0.861, 0.939, and 0.886 in the training dataset (Figure 5C), which were statistically significant. The expression of BCL2L1, CDK1, HSP90AA1, IL-2, and TERT was discovered to be significant in accordance with the survival of CHD patients following univariate Cox regression analyses (Figure 6A). The outcomes of multivariate Cox regression analysis demonstrated that the CDK1, HSP90AA1, and TERT were still independent prognostic predictors for CHD patients after correcting various confounding factors (Figure 6B). Furthermore, to predict 1-, 3-, and 5-year survival probability of CHD patients, a nomogram was established by utilizing TERT and the outcomes demonstrated that the expression of TERT affects the survival probability of CHD patients significantly (Figure 6C). The good calibration of the model was later confirmed by the calibration plot which displayed brilliant accordance between the nomogram-predicted survival probability and actual observed results (Figure 6D).

## Discussion

Coronary heart disease has been recognized as one of the dominant global causes of death. Coronary heart disease (CHD) is a group of diseases with many forms including myocardial infarction, angina pectoris, ischemic cardiomyopathy, asymptomatic, and sudden cardiac death. Constant factors (such as age and gender) and variable factors (such as dyslipidemia, hypertension, diabetes, and smoking) interact together and become causes of coronary heart disease (29). At the same time, coronary heart disease will affect the patient's systemic multiple systems. Its influence is not limited to the heart, but also affects lung function, skeletal muscle function, activity ability, mental state, etc. There are many ways to treat coronary heart disease. Drug therapy is the basis. Nitrates have a long history as a remedy for angina (30). However, because of its failure to reduce hard end points such as mortality, it is only used to relieve symptoms in angina pectoris. Calcium channel blockers are unique in alleviating coronary artery spasm, while beta receptor blockers play an important role in alleviating fatigue angina and reducing cardiac events (31, 32). The role of antiplatelet agents in the prevention of ischemic events and mortality in coronary heart disease has been demonstrated (33). Anticoagulant and thrombolytic drugs are also being used gradually. The lipid-regulating effects of statins, such as endothelium protection, anticoagulation, and anti-inflammation, have also become hot issues in this field (34, 35). Percutaneous coronary intervention is also a revolutionary progress in the field of coronary heart disease treatment (36, 37). The research of surgical treatment of coronary heart disease has experienced nearly 100 years of exploration and has put forward many different surgical methods and surgical techniques, according to the breakthrough progress, can be divided into three stages: surgical intervention of the body physiological treatment of coronary heart disease, focusing on increasing collateral circulation, and directly increasing myocardial blood supply (38).

Polysaccharide derivatives such as cellulose derivatives have attracted increasing attention due to their relative abundance and ability to maintain drug release. Rossana B Simeoni et al. explored the possibility of using bacterial cellulose (BC) membrane patches containing co-cultured cells to limit post-myocardial infarction pathology (39, 40). The results of the study leaded by Sahar S Abd-Elhalem et.al. suggested that nanofibrillation cellulose-loaded methotrexate may improve renal function tests, markers of renal tissue inflammation, and fibrosis (41). In addition, nanofibrillated cellulose carriers maintain long-term slow-release of methotrexate, making it more likely than nanosilica to play a role in further medical applications as an effective new drug carrier with minimal side effects on leukemia model kidney tissue (42). At present, an increasing number of scientists focus on exploring the functional rules of polysaccharides and their related derivatives in the treatment of cardiovascular diseases. The development of cellulose-based nano-DDs for intravenous or oral applications is about to become an essential research field and lead to more commercial standings in the market. Nanocellulose can be used as a carrier to transport antiplatelet drugs and anticoagulant drugs into the body and achieve the purpose of sustained release or even selective action on the lesion site, so as to reduce other cerebrovascular events caused by drug side reactions. However, due to the fact that cellulose is an exogenous substance in the human body, few studies on the mechanism of cellulose and human body have been published, and relevant clinical trials are even rare. Our study is the first analysis to explore the relevant target of cellulose action in patients with coronary heart disease through network pharmacology, providing some molecular basis for subsequent materials science, basic medicine, and clinical medicine researchers. We obtained 92 genes associated with cellulose and coronary heart disease through the intersection of different databases. Then, we found 10 key genes through network analysis: HRAS, STAT3, HSP90AA1, FGF2, VEGFA, CXCR4, TERT, IL2, BCL2L1, and CDK1. Molecular docking of the 10 genes revealed their association with the relevant receptors. Through KEGG and GO analysis, we found that these related genes are more enriched in metabolism- and activation-related functions, such as calcium signaling pathway and cAMP signaling pathway. This further confirmed that cellulose polysaccharides can also interact with cardiovascular disease as a molecule. With the aim of onwards exploring the impact of these key genes on the prognosis of patients, we screened six genes that were more correlated with the prognosis to construct a prognosis model. Patients with high expression of CDK1 have a lower survival rate. Patients with high expression of HSP90AAI have a better survival rate. Patients with high expression of CXCR4 have a lower survival rate. Patients with high expression of IL2 have a lower survival rate. Patients with high expression of VEGFA have a lower survival rate. Patients with high expression of TERT have a lower survival rate. According to the AUC curve, the prognostic model had a good predictive effect and was statistically significant.

Our study is the first study to explore the interacting targets of cellulose and CHD with the construction of a prognostic signature. Our findings provide insights for future molecular design, drug development, and clinical trials. However, our study still has limitations, for instance, we did not conduct *in vitro* and *in vivo* experiments to verify the capabilities of relevant targets. At present, cellulose modified to be used in the treatment of diseases is still a long way to go. The research on this application in medical materials science and pharmacology is not deep enough, and interdisciplinary research should be further proposed. More animal studies need to be conducted to explore potential safety and efficacy metrics. Therefore, more efforts are needed in this field.

## Data availability statement

The original contributions presented in the study are included in the article/supplementary material, further inquiries can be directed to the corresponding author/s.

## Author contributions

LL, YZ, and HL completed data download, data collation, and data analysis. LL and YD created the charts. MW organized the figures. LL and JL completed the writing of Introduction and Discussion and carefully revised the article and finished editing the paper. YZ wrote the rest of the article. All authors contributed to the article and approved the submitted version.

## Funding

This study is supported by research on the role and mechanism of TLR4 signaling pathway in the development of anxiety induced coronary heart disease, Hubei Health Commission, WJ2021F061, 2021.01-2022.12.

## Conflict of interest

The authors declare that the research was conducted in the absence of any commercial or financial relationships that could be construed as a potential conflict of interest.

## Publisher's note

All claims expressed in this article are solely those of the authors and do not necessarily represent those of their affiliated organizations, or those of the publisher, the editors and the reviewers. Any product that may be evaluated in this article, or claim that may be made by its manufacturer, is not guaranteed or endorsed by the publisher.

## References

[1] Wirtz PH von Känel R. Psychological stress, inflammation, and coronary heart disease. Curr Cardiol Rep. (2017) 19:111. 10.1007/s11886-017-0919-x 28932967

[2] Shah B Newman JD Woolf K Ganguzza L Guo Y Allen N. Anti-inflammatory effects of a vegan diet vs. the American heart association-recommended diet in coronary artery disease trial. J Am Heart Assoc. (2018) 7:e011367. 10.1161/JAHA.118.011367 30571591 PMC6405545

[3] Tian Y Deng P Li B Wang J Li J Huang Y. Treatment models of cardiac rehabilitation in patients with coronary heart disease and related factors affecting patient compliance. Rev Cardiovasc Med. (2019) 20:27–33. 10.31083/j.rcm.2019.01.53 31184093

[4] Katta N Loethen T Lavie CJ Alpert MA. Obesity and coronary heart disease: epidemiology, pathology, and coronary artery imaging. Curr Probl Cardiol. (2021) 46:100655. 10.1016/j.cpcardiol.2020.100655 32843206

[5] Stewart RAH Held C Hadziosmanovic N Armstrong PW Cannon CP Granger CB. Physical activity and mortality in patients with stable coronary heart disease. J Am Coll Cardiol. (2017) 70:1689–700. 10.1016/j.jacc.2017.08.017 28958324

[6] Bačáková L Novotná K Parízek M. Polysaccharides as cell carriers for tissue engineering: the use of cellulose in vascular wall reconstruction. Physiol Res. (2014) 63:S29–47. 10.33549/physiolres.932644 24564664

[7] Zhang J Liu Y Tang Q Zhou S Feng J Chen H. Polysaccharide of ganoderma and its bioactivities. Adv Exp Med Biol. (2019) 1181:107–34. 10.1007/978-981-13-9867-4_4 31677141

[8] Jin M Shi J Zhu W Yao H Wang DA. Polysaccharide-based biomaterials in tissue engineering: a review. Tissue Eng Part B Rev. (2021) 27:604–26. 10.1089/ten.teb.2020.0208 33267648

[9] Nai J Zhang C Shao H Li B Li H Gao L. Extraction, structure, pharmacological activities and drug carrier applications of *Angelica sinensis* polysaccharide. Int J Biol Macromol. (2021) 183:2337–53. 10.1016/j.ijbiomac.2021.05.213 34090852

[10] Chen L Huang G. Antitumor activity of polysaccharides: an overview. Curr Drug Targets. (2018) 19:89–96. 10.2174/1389450118666170704143018 28676001

[11] Su J Jiang L Wu J Liu Z Wu Y. Anti-tumor and anti-virus activity of polysaccharides extracted from *Sipunculus nudus* (SNP) on Hepg2215. Int J Biol Macromol. (2016) 87:597–602. 10.1016/j.ijbiomac.2016.03.022 26987430

[12] Liu C Cui Y Pi F Cheng Y Guo Y Qian H. Extraction, purification, structural characteristics, biological activities and pharmacological applications of acemannan, a polysaccharide from *Aloe vera*: a review. Molecules. (2019) 24:1554. 10.3390/molecules24081554 31010204 PMC6515206

[13] Wang J Tavakoli J Tang Y. Bacterial cellulose production, properties and applications with different culture methods - A review. Carbohydr Polym. (2019) 219:63–76. 10.1016/j.carbpol.2019.05.008 31151547

[14] Hu Y Wang Y An F Dai N. The efficacy of double-heart nursing in combination with seaweed polysaccharide for patients with coronary heart disease complicated with diabetes: a pilot, randomized clinical trial. Dis Markers. (2022) 2022:2159660. 10.1155/2022/2159660 35419116 PMC9001089

[15] Fernandes IAA Pedro AC Ribeiro VR Bortolini DG Ozaki MS Maciel GM. Bacterial cellulose: from production optimization to new applications. Int J Biol Macromol. (2020) 164:2598–611. 10.1016/j.ijbiomac.2020.07.255 32750475

[16] Islam SU Ul-Islam M Ahsan H Kamal T Ahmad M Abbas Y. Potential applications of bacterial cellulose and its composites for cancer treatment. Int J Biol Macromol. (2021) 168:301–9. 10.1016/j.ijbiomac.2020.12.042 33316340

[17] Buldum G Mantalaris A. Systematic understanding of recent developments in bacterial cellulose biosynthesis at genetic, bioprocess and product levels. Int J Mol Sci. (2021) 22:7192. 10.3390/ijms22137192 34281246 PMC8268586

[18] Tang J Sisler J Grishkewich N Tam KC. Functionalization of cellulose nanocrystals for advanced applications. J Colloid Interface Sci. (2017) 494:397–409. 10.1016/j.jcis.2017.01.077 28187295

[19] Picheth GF Pirich CL Sierakowski MR Woehl MA Sakakibara CN de Souza CF. Bacterial cellulose in biomedical applications: a review. Int J Biol Macromol. (2017) 104(Pt A):97–106. 10.1016/j.ijbiomac.2017.05.171 28587970

[20] Lugoloobi I Maniriho H Jia L Namulinda T Shi X Zhao Y. Cellulose nanocrystals in cancer diagnostics and treatment. J Control Release. (2021) 336:207–32. 10.1016/j.jconrel.2021.06.004 34102221

[21] Du H Liu W Zhang M Si C Zhang X Li B. Cellulose nanocrystals and cellulose nanofibrils based hydrogels for biomedical applications. Carbohydr Polym. (2019) 209:130–44. 10.1016/j.carbpol.2019.01.020 30732792

[22] Rana AK Frollini E Thakur VK. Cellulose nanocrystals: pretreatments, preparation strategies, and surface functionalization. Int J Biol Macromol. (2021) 182:1554–81. 10.1016/j.ijbiomac.2021.05.119 34029581

[23] Lucia A Bacher M van Herwijnen HWG Rosenau T A. direct silanization protocol for dialdehyde cellulose. Molecules. (2020) 25:2458. 10.3390/molecules25102458 32466232 PMC7287999

[24] Sun Y Chu Y Wu W Xiao H. Nanocellulose-based lightweight porous materials: a review. Carbohydr Polym. (2021) 255:117489. 10.1016/j.carbpol.2020.117489 33436249

[25] Curvello R Raghuwanshi VS Garnier G. Engineering nanocellulose hydrogels for biomedical applications. Adv Colloid Interface Sci. (2019) 267:47–61. 10.1016/j.cis.2019.03.002 30884359

[26] Dai L Si C. Recent advances on cellulose-based nano-drug delivery systems: design of prodrugs and nanoparticles. Curr Med Chem. (2019) 26:2410–29. 10.2174/0929867324666170711131353 28699504

[27] Liu H Zhuang J Tang P Li J Xiong X Deng H. The role of the gut microbiota in coronary heart disease. Curr Atheroscler Rep. (2020) 22:77. 10.1007/s11883-020-00892-2 33063240

[28] Colić M Tomić S Bekić M. Immunological aspects of nanocellulose. Immunol Lett. (2020) 222:80–9. 10.1016/j.imlet.2020.04.004 32278785

[29] Carney RM Freedland KE. Depression and coronary heart disease. Nat Rev Cardiol. (2017) 14:145–55. 10.1038/nrcardio.2016.181 27853162

[30] Malakar AK Choudhury D Halder B Paul P Uddin A Chakraborty S. Review on coronary artery disease, its risk factors, and therapeutics. J Cell Physiol. (2019) 234:16812–23. 10.1002/jcp.28350 30790284

[31] Wang L Ai D Zhang N. Exercise benefits coronary heart disease. Adv Exp Med Biol. (2017) 1000:3–7. 10.1007/978-981-10-4304-8_1 29098612

[32] ALLHAT Officers and Coordinators for the ALLHAT Collaborative Research Group. The antihypertensive and lipid-lowering treatment to prevent heart attack trial. Major outcomes in high-risk hypertensive patients randomized to angiotensin-converting enzyme inhibitor or calcium channel blocker vs. diuretic: the Antihypertensive and Lipid-Lowering Treatment to Prevent Heart Attack Trial (ALLHAT). JAMA. (2002) 288:2981–97. 10.1001/jama.288.23.2981 12479763

[33] Bhatt DL Steg PG Mehta SR Leiter LA Simon T Fox K. Ticagrelor in patients with diabetes and stable coronary artery disease with a history of previous percutaneous coronary intervention (THEMIS-PCI): a phase 3, placebo-controlled, randomized trial. Lancet. (2019) 394:1169–80. 10.1016/S0140-6736(19)31887-2 31484629

[34] Agrawal H Choy HK Liu J Auyoung M Albert MA. Coronary artery disease. Arterioscler Thromb Vasc Biol. (2020) 40:e185–92. 10.1161/ATVBAHA.120.313608 32579480

[35] Taguchi I Iimuro S Iwata H Takashima H Abe M Amiya E. High-dose versus low-dose Pitavastatin in Japanese Patients With Stable Coronary Artery Disease (REAL-CAD): a randomized superiority trial. Circulation. (2018) 137:1997–2009. 10.1161/CIRCULATIONAHA.117.032615 29735587 PMC5959207

[36] Bhatt DL. Percutaneous coronary intervention in 2018. JAMA. (2018) 319:2127–28. 10.1001/jama.2018.5281 29800163

[37] Case BC Waksman R. Coronary heart disease: have we reached a plateau in primary prevention? J Am Heart Assoc. (2020) 9:e04963. 10.1161/JAHA.120.016034 32237973 PMC7428609

[38] Stone GW Kappetein AP Sabik JF Pocock SJ Morice MC Puskas J. Five-year outcomes after PCI or CABG for left main coronary disease. N Engl J Med. (2019) 381:1820–30. 10.1056/NEJMoa1909406 31562798

[39] Simeoni RB Mogharbel BF Francisco JC Miyague NI Irioda AC Souza CM. Beneficial roles of cellulose patch-mediated cell therapy in myocardial infarction: a preclinical study. Cells. (2021) 10:424. 10.3390/cells10020424 33671407 PMC7922134

[40] Bitterman LA Martinez A Mulholland C Somerville T Prieto-Centurion D Zodrow KR. Bacterial cellulose spheres that encapsulate solid materials. J Vis Exp. (2021) e62286. 10.3791/62286 33720144

[41] Abd-Elhalem SS El-Shinnawy NA Abu-El Magd EE El Zawawy WK Haggag NZ. Application of either nano fibrillated cellulose methotrexate or nano silicon dioxide methotrexate composites against renal fibrosis in leukemia rat model. Int J Biol Macromol. (2020) 157:329–39. 10.1016/j.ijbiomac.2020.04.110 32330502

[42] Ilves M Vilske S Aimonen K Lindberg HK Pesonen S Wedin I. Nanofibrillated cellulose causes acute pulmonary inflammation that subsides within a month. Nanotoxicology. (2018) 12:729–46. 10.1080/17435390.2018.1472312 29848128

